# Complete mitochondrial genome of the hard clam (*Mercenaria mercenaria*)

**DOI:** 10.1080/23802359.2019.1681306

**Published:** 2019-10-24

**Authors:** Zhi Hu, Hao Song, Cong Zhou, Zheng-Lin Yu, Mei-Jie Yang, Tao Zhang

**Affiliations:** a CAS Key Laboratory of Marine Ecology and Environmental Sciences, Institute of Oceanology, Chinese Academy of Sciences, Qingdao, Shandong, China;; b Laboratory for Marine Ecology and Environmental Science, Qingdao Pilot National Laboratory for Marine Science and Technology, Qingdao, Shandong, China;; c University of Chinese Academy of Sciences, Beijing, China;; d Center for Ocean Mega-Science, Chinese Academy of Sciences, Qingdao, Shandong, China

**Keywords:** *Mercenaria mercenaria*, Venreidae, mitogenome

## Abstract

The hard clam (*Mercenaria mercenaria*) is an important economic and ecological bivalve. In this study, the mitochondrial genome was sequenced. The sequenced genome size was 18,360 bp. The nucleotide composition was asymmetric with a AT bias. Mitogenome contained 13 protein-coding genes (PCGs), 2 rRNA genes, and 22 tRNA genes. Of 13 PCGs, 3 genes (*cox3*, *nad3*, and *cox2*) had incomplete stop codons. Furthermore, phylogenetic analysis using 12 PCGs (except *atp8*) figured out that *M. mercenaria* was closely related to genus *Dosinia*. The complete mitogenome of *M. mercenaria* provides essential information for further phylogenetic and evolutionary analysis in Veneridae.

Veneridae presumably has more than 800 species and is a large and diverse family of Bivalvia (Mikkelsen et al. 2006), many of which are commercially important in benthic communities (Canapa et al. 2003). The hard clam (*Mercenaria mercenaria*) is a native kind of bivalve on the east coast of the US and Canada (Menzel 2010). Fu-Sui Zhang imported hard clams from America to China in 1997. Because of its strong life, it became an important cultured bivalve in China. The mitogenome has been suggested to be a good source for molecular biology because of rapid evolutionary rate and lack of recombination (He et al. 2011). In this paper, we report the complete mitochondrial genome of the hard clam *M. mercenaria* to better understand the phylogenetic position within the Veneridae family.

The adult hard clam was collected from Dongying, Shandong province, China (37.25 N 118.55 E) and then cultured by the Mashan Group Co., Ltd. in Shandong Province. During culture, the hard clam was acclimated to the seawater (25 °C, 30% salinity) under continuous aeration and fed with *Isochrysis galbana*. The adductor muscle was sequenced. Specimen (Collection Number: MBM286619) was deposited in the museum of Institute of Oceanology, Chinese Academy of Sciences. The complete mitogenome of *M. mercenaria* was 18,360 bp in length (GeneBank Accession: MN233789), which was within the range of genome sizes for already sequenced molluscan mitogenomes. The mitogenome size was similar to *Dosinia* clam and length differences were mostly due to the size variations of the non-coding region (Lv et al. 2018). The overall base composition was 27.17% A, 21.62% G, 9.44% C, and 41.77% T. The GC content was 31.06%. The mitogenome contained 13 protein-coding genes (PCGs), 2 ribosomal RNA genes, 22 transfer RNA genes, and a control region. *Atp8* gene was found in the mitogenome, which has been reported as missing in several bivalve species (He et al. 2011). Six PCGs (*cox3*, *nad2*, *nad4l*, *nad4*, *atp6*, and *cox2*) used ATG as initiation codon. Four PCGs (*nad1*, *cob*, *nad3*, and *nad5*) used ATT as an initiation codon. *nad6* and *cox1* used ATA as initiation codon and *atp8* used ATC as initiation codon. Most PCGs used TAG or TAA as stop codon. Incomplete stop codons in *cox3*, *nad3*, and *cox2* were found as T(aa) and TA(a), these incomplete termination codons might be completed as TAA by post-transcriptional polyadenylation (Ojala et al. 1981).

The other 14 clams of family Veneridae mitogenome sequences in public were used in phylogenetic analysis. *Solen grandis* and *Solen strictus* were used as outgroups. We used MEGA 7 (Kumar et al. 2016) to construct the phylogenetic relationships of the *M. mercenaria* and related Veneridae by neighbour-joining method with 1000 bootstrap replicates based on the 12 PCGs (except *atp8*) (Figure 1). The resultant phylogenetic tree indicated that *M. mercenaria* was closely related to genus *Dosinia*. The complete mitogenome of *M. mercenaria* provides essential information for further phylogenetic and evolutionary analysis in Veneridae.

**Figure 1.**
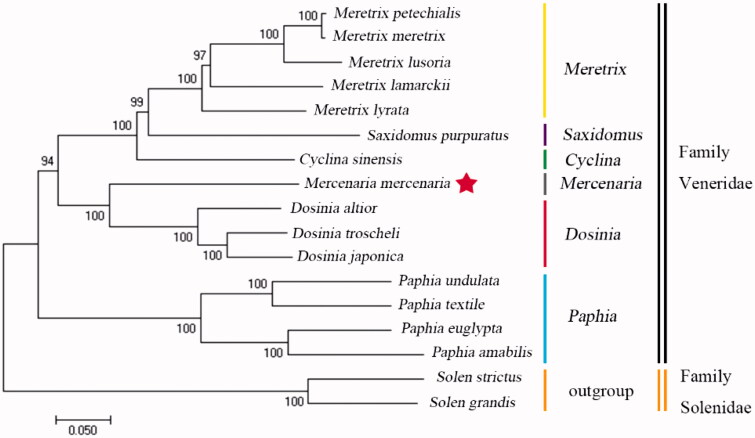
Phylogenetic trees based on the nucleotide sequences of 12 concatenated protein-coding genes. The numbers in the phylogenetic tree are the bootstrap probability values. Vertical stick indicates genus, double vertical sticks indicates families. The genome sequence in this study is labelled with a red star. GenBank accession numbers of the sequences were used for the tree as follows: *Meretrix petechialis* (EU145977); *Meretrix meretrix* (GQ463598); *Meretrix lusoria* (GQ903339); *Meretrix lamarckii* (GU071281); *Meretrix lyrate* (KC832317); *Saxidomus purpuratus* (KP419933); *Cyclina sinensis* (KU097333); *Dosinia altior* (MG543473); *Dosinia troscheli* (MG543474); *Dosinia japonica* (MF401432); *Paphia undulata* (JF969278); *Paphia textile* (JF969277); *Paphia euglypta* (GU269271); *Paphia amabilis* (JF969276); *Solen strictus* (JN786377); *Solen grandis* (HQ703012).
